# Identification of uranium signatures relevant for nuclear safeguards and forensics

**DOI:** 10.1007/s10967-017-5247-5

**Published:** 2017-04-20

**Authors:** Zsolt Varga, Judit Krajkó, Maxim Peńkin, Márton Novák, Zsuzsanna Eke, Maria Wallenius, Klaus Mayer

**Affiliations:** 1 European Commission, Directorate for Nuclear Safety and Security, Joint Research Centre, Postfach 2340, 76125 Karlsruhe, Germany; 2 Department of Safeguards, International Atomic Energy Agency, Vienna International Centre, P.O. Box 100, 1400 Vienna, Austria; 3 Joint Research and Training Laboratory on Separation Techniques (EKOL), Eötvös Loránd University, Pázmány Péter sétány 1/A, 1117 Budapest, Hungary; 4 Wessling International Research and Educational Center, Fóti út 56, 1047 Budapest, Hungary

**Keywords:** Uranium, Uranium ore concentrate, Nuclear safeguards, Nuclear forensics, Elemental impurities, Isotopic composition

## Abstract

The paper describes the applicability of different characteristics (signatures) in nuclear safeguards and forensics for assessment of uranium material provenance in terms of production process. The study follows a uranium ore concentrate production from an ore to a U_3_O_8_ product. It turned out that rare-earth elemental pattern, radiochronometry (age of ore body and material production date), sulphur and organic impurities are useful to find out the origin or history of the material, while certain trace-elements and isotopics of Pb or Sr were found to be inconclusive. The results will be important to understand the signatures in nuclear safeguards and forensics.

**Electronic supplementary material:**

The online version of this article (doi:10.1007/s10967-017-5247-5) contains supplementary material, which is available to authorized users.

## Introduction

In nuclear safeguards and nuclear forensics, several characteristics of the material in question are used either to verify the declared origin (safeguards) or to identify the source of an unknown nuclear material (nuclear forensics) [1–3]. These characteristics involve, for instance, concentrations of major, minor and trace-level constituents, isotopic composition of Pb, Sr or S impurities, material morphology or molecular structure [4–6]. Using this complex set of parameters, the origin of a sample in question can be verified or identified with higher confidence. However, as several types of technologies for ore leaching, uranium extraction and purification exist with various types of feed materials (e.g. uranium ores of different mineralogical nature or secondary sources), the interpretation of such characteristics (also called signatures) are in most cases very difficult, as they can be both be dominated by the feed material or altered by the process and the regents used. In consequence, certain measurable characteristics do not serve as useful signature due to their high variability.

**Fig. 1:**
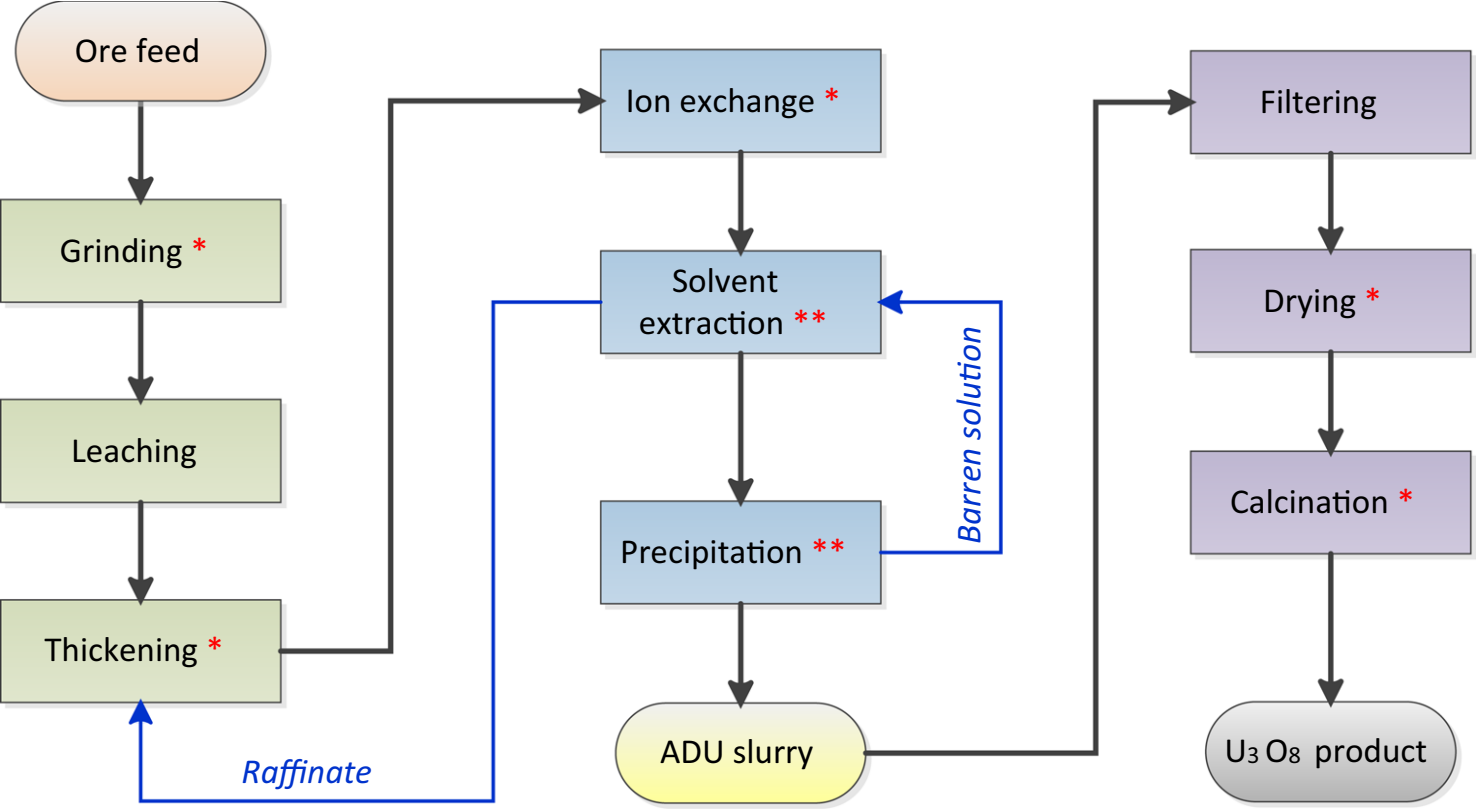
Schematic of the process flow. The *red asterisks* indicate those process stages with outgoing streams which were sampled. (Color figure online)

This study follows a uranium ore concentrate (UOC) production route from quartz-pebble conglomerate ore feed to the calcined U_3_O_8_ powder product at an undisclosed facility in South Africa. The ore is leached with concentrated sulphuric acid, filtered, and then uranium is purified by ion exchange (IX) and solvent extraction (SX) with tri-octyl/dodecyl amine. The precipitated ammonium diuranate (ADU) intermediate product is dried and transported for calcination into U_3_O_8_. The samples used in this study, as provided by the plant operators, are listed in Table 1. The schematic of the process flow is shown in Fig. 1. The samples collected are believed to represent a flow of material originating from the same feed. The sampling was arranged by the RSA Support Programme through task A 1790. A subset of ten samples was provided by the IAEA Department of Safeguards to JRC Karlsruhe in April 2013 for analysis under the European Commission Support Programme task A 1753.

**Table 1:** List of samples

Sample	Type of sample	Approximate U content measured (μg g^−1^)
Dried ore slurry	Solid	300
Cleared leachate solution	Liquid	178
Ion exchange eluate	Liquid	3210
Loaded solution for SX (OK liquor)	Liquid	9054
Raffinate	Liquid	248
ADU slurry-1	Slurry	128,000
Barren mother liquor	Liquid	2.77
ADU slurry-2	Slurry	261,800
Dried ADU powder	Solid	634,700
Calcined U_3_O_8_ powder	Solid	748,000

The aim of the present study is to assess variation of several characteristics (signatures) from an industrial ore mining, uranium concentration to the final purified U_3_O_8_ product. The purpose is to identify relevant characteristics, which can be used to establish or verify the type of feed ore and/or the process employed, ultimately pointing at the ore type and concentration and purification processes used. By this means the relative significance of signatures can also be assessed, which is valid only for the current case study using these steps. However, several conclusions can also be generalized to focus on the promising signatures or to discard the excessive or inefficient characteristics.

## Experimental

### Reagents and materials

All labware was thoroughly cleaned before use. Suprapur grade hydrofluoric and nitric acids (Merck, Darmstadt, Germany) were used for the sample preparation. HNO_3_ was further purified by subboiled distillation (AHF Analysentechnik AG, Germany). For dilutions ultrapure water was used (Elga LabWater, Celle, Germany). A ^233^U isotopic reference material was used to spike the samples for the uranium concentration measurements by mass spectrometry with isotope dilution. The ^233^U concentration in the spike was calibrated against EC NRM 101 uranium metal by thermal ionization mass spectrometry (TIMS). A custom-made natural thorium solution from Spex Certiprep Inc. (Metuchen, USA) at a Th concentration of 1000 μg g^−1^ was used as a spike for the ^230^Th isotope dilution measurements. Uranium U-010 standard reference material (nominally 1% ^235^U) from National Bureau of Standards (USA) was used to correct for instrumental mass discrimination. Isotopic reference materials IRMM-035 (certified *n*(^230^Th)/*n*(^232^Th) is (1.1481 ± 0.0078) × 10^−5^) and IRMM-185 (certified *n*(^235^U)/*n*(^238^U) is (2.00552 ± 0.00060) × 10^−2^) were used to check the accuracy of the thorium and uranium isotope ratio measurements, respectively.

TRU^®^ (octylphenyl-*N*,*N*-di-isobutyl carbamoyl phosphine oxide in TBP, rare-earth elements measurements), TEVA^®^ (Aliquat-336, Th measurements) and LN^®^ (di(2-ethylhexyl)orthophosphoric acid, Nd measurements) extraction chromatographic resins (50–100 μm particle size) supplied by Triskem International (Bruz, France) were used and covered by porous Teflon frits to avoid mixing (Reichelt Chemietechnik Heidelberg, Germany). Before use, the resin was cleaned and conditioned. For S measurements, the anion exchange resin (AG 1-X4, Cl^−^ form, 100–200 mesh, Bio-Rad Laboratories, USA) was used. The separation and measurement are discussed in details elsewhere [5–8]. The Nd measurements were performed in a two-step procedure: first the rare-earth elements (REE) were separated; then LN resin was applied to obtain the Nd fraction. Further details on the separation and measurement can be found elsewhere [9].

Helium gas (purity: 99.999%) and SupraSolv^®^ n-hexane (Merck KGaA, Darmstadt, Germany) were used for the GC–MS measurement.

### Investigated samples and process description

The list of investigated samples is shown in Table 1.

The sample sizes were relatively large, above 50 g each. They were assumed to be representative of the processed sampled feed material, though deviation could occur due to e.g. difficulty to follow the material flow versus process timeline, cross-contamination or equipment corrosion/erosion. The homogeneity of the sample was varying: especially the liquid samples from the early stages of the process (e.g. cleared leachate) contained significant amount of solid residues. Although part of the solid residue was assumed to be separated from the liquid phase during the process (e.g. the ion exchange resin acts also as a filter to remove solid particulates), it cannot be excluded that the solid residue contributed to the total impurity content.

### Analytical measurements

From each sample, about 100–500 mg aliquot was taken. The liquid samples were stirred, left for 2 h to settle, then the supernatant (liquid phase) was only used for the forthcoming analyses. The solid samples were dissolved in 8 M HNO_3_/0.02 HF (for impurity analysis and U isotopic measurements), 8 M HNO_3_ only (for Sr, Pb, Nd, Th isotopics) and H_2_O leach (for S isotopics). Further details can be found elsewhere [5, 9–11]. Th isotopic analyses and U, Th and impurity concentration measurements were carried out using a double-focusing magnetic sector inductively coupled plasma mass spectrometer (ICP-MS) equipped with a single electron multiplier (Element2, Thermo Electron Corp., Bremen, Germany). All measurements were carried out in a low resolution mode (*R* = 300) using a low-flow micro-concentric nebulizer operated in a self-aspirating mode (flow rate was approximately 50 μL min^−1^) in combination with a Teflon Scott-type spray chamber.

For impurity measurements a sample aliquot was diluted gravimetrically to concentration about 100 μg U g^−1^, and analysed using Rh internal standard with matrix-matched calibration [4]. The compiled results are summarized in Supplement 1.

For those samples which contain low REE concentrations (ADU powder and U_3_O_8_), REE analysis was accomplished after chemical separation of the REE group [7].

The U, Pb, Sr and S isotopic measurements were performed on a NuPlasma™ (NU Instruments, Oxford, United Kingdom) double-focusing multi-collector inductively coupled plasma mass spectrometer (MC-ICP-MS), equipped with 11 Faraday collectors and 3 discrete dynode electrode multipliers. The instrument was operated in a low mass resolution mode (*R* = 300). The sample solutions were introduced into the plasma using a low-flow Teflon microconcentric nebulizer operated in a self-aspirating mode in combination with a desolvation unit (DSN-100, NU Instruments, Oxford, United Kingdom). Further details can be found elsewhere [5, 10].

The organic residue content of the ADU powder sample was analysed by gas chromatography mass spectrometry (GC–MS) after extracting with hexane containing 0.1 M NH_4_NO_3_ to test if any typical solvent extractants used for uranium processing could be detected. The target organic constituents were tributyl phosphate (TBP), trioctylphosphine oxide (TOPO) and tri-*n*-octylamine (TNOA). The analysis was performed on an Agilent 6890 N GC coupled to a 5973 inert MS with an EI ion source (Agilent, Santa Clara, CA, USA). To improve detection limits a Gerstel CIS4 programmed temperature vaporization inlet system (Gerstel GmbH, Mülheim an der Ruhr, Germany) was used to enable large volume injection in solvent vent mode (150 μL; injection speed: 1.35 μL s^−1^, vent flow: 150 mL min^−1^). Initially, the temperature of the inlet was set to 10 °C and held for 1.85 min then increased to 70 °C at a rate of 12 °C s^−1^ after holding it for 3 min it was further increased to 320 °C at a rate of 12 °C s^−1^. This final temperature was held for 10 min. Then it was increased further to 320 °C at a rate of 12 °C s^−1^ and kept at this final temperature for 10 min. A 30 m long DB-5MS UI capillary column with 0.25 mm inner diameter and 0.25 μm film thickness was used for separation. Helium was used as carrier gas with a constant flow rate of 1.0 mL min^−1^. The GC oven temperature was maintained at 40 °C for 6.75 min, increased to 310 °C at a rate of 25 °C min^−1^ until 180 °C followed by a heating a rate of 20 °C min^−1^. The final oven temperature was held for 10 min. To control the performance of the extraction and the large volume injection acenaphtene-d10, phenantrene-d10, chrysene-d12 were used.

## Results and discussion

### Step 1: Leaching of the ore

The feed ore is milled to powder before it enters the uranium concentration plant as thick slurry (pulp) and is leached with sulphuric acid to solubilize uranium. As the ore has already been contacted with water before the sulphuric acid leach, it can be assumed that the water-soluble constituents (e.g. salts of alkali metals) have already been removed, to a great degree, from the slurry solid phase. In the initial process description it was not mentioned if an oxidizer (MnO_2_ or O_2_) was used to accelerate the leaching. The leaching conditions were stated as 18 h at 50–60 °C. The cleared leachate was sampled at the facility.

The results of the impurity measurements are summarized in Supplement 1. The impurity content of the ore sample was measured after total dissolution using HF/HNO_3_ acid mixture. However, in order to simulate the plant process, the ore sample was also leached at JRC-Karlsruhe with diluted sulphuric acid. The conditions for the sulphuric acid leaching were: 10% H_2_SO_4_, 1:1 solid-to-liquid ratio, 24 h leaching time, followed by centrifugation and filtering through a 0.45 μm cellulose-acetate membrane filter. The leachate sample was measured as received, i.e. using an aliquot of the supernatant. Note that for the low uranium content samples the impurity concentrations in Supplement 1 are given as microgram analyte per gram of sample (and are not normalized to the uranium content). However, for the interpretation of the results it was necessary to use concentration values normalized to the uranium content. The concentration ratio of the elements in the ore measured after total dissolution relative to the cleared leachate sample, as well as the concentration ratio in the “in-house” leachate relative to that of the cleared leachate sample (after all values were normalized to the uranium content) are given in Table 2.

**Table 2:** Variation of impurity concentrations during ore leaching expressed as ratios of concentrations normalized to U

	Ratio ore total versus cleared leachate	Highly affected	In-house leach versus cleared leachate		Ratio ore total versus cleared leachate	Highly affected	In-house leach versus cleared leachate
Al	24	**X**	1.3	Nb	584	**X**	45
As	2.8	**X**	0.7	Nd	7.2	**X**	1.4
Ba	6600	**X**	28	Ni	1.5		0.6
Bi	31	**X**	32	P			2.0
Ca	2.3	**X**	3.0	Pb	21	**X**	4.6
Cd	2.3	**X**	0.2	Pd	1.4		1.0
Ce	11	**X**	1.5	Pr	8.8	**X**	1.5
Co	2.0	**X**	0.5	Rb	68	**X**	1.2
Cr	6.9	**X**	1.3	Re			1.3
Cs	9.0	**X**	1.8	S	0.8		32
Cu	3.4	**X**	0.9	Sb	14	**X**	1.3
Dy	1.2		1.0	Sc	7.5	**X**	1.1
Er	1.3		1.0	Se			2.0
Eu	3.3	**X**	1.1	Si	98	**X**	1.1
Fe	4.3	**X**	1.2	Sm	3.3	**X**	1.2
Ga	34	**X**	1.8	Sr	11	**X**	1.5
Gd	2.3	**X**	1.1	Tb	1.5		1.0
Hf	105	**X**	4.4	Th	1.2		1.0
Ho	1.2		1.0	Ti	646	**X**	41
K	43	**X**	0.9	Tl			1.0
La	14	**X**	1.7	Tm	1.3		0.9
Li			1.1	V	15	**X**	1.1
Lu	1.6		1.0	Y	1.2		1.0
Mg	2.8	**X**	1.2	Yb	1.4		1.0
Mn	0.18	*X*	0.2	Zn	2.1	**X**	0.2
Mo	38	**X**	8.5	Zr	695	**X**	25
Na			0.3				

The highly affected elements, where the ratio of the analyte concentration in the ore measured after total dissolution versus the analyte concentration in the cleared leachate are higher than 2 (arbitrarily chosen), are indicated. Unlike all other impurities, the Mn concentration in the leachate is much higher than in ore. U concentration in the cleared leachate was (178.1 ± 2.3) μg g^−1^

The most important conclusion is that the ore leaching using sulphuric acid significantly affects the impurity pattern: such leaching is known to be almost quantitative for uranium; however, for most of the other elements the sulphuric acid leaching is not effective. Thus the total dissolution highly overestimates the impurity content, which actually will be present in the industrial process solution after the leaching step. For most elements, only 1–40% of their content can be dissolved and recovered by sulphuric acid leach (indicated in Supplement 1), their relative concentrations to U are also altered by the sulphuric acid leach. The change of the elemental pattern by the leaching process highly impedes the interpretation of the impurity results and suggests that the ore elemental composition results measured after complete dissolution of the samples shall be very different from the impurity results obtained by the sulphuric acid leaching. The most important reasons behind this effect (so-called fractionation), relating to the ore composition, are as follows:

1. Presence of less-soluble minerals: possibly this is the dominant factor for several transition metals, such as Hf, Ti, Nb or Zr, which are generally present as refractory oxides.
2. Presence of silicates; this results in an incomplete recovery of, for instance, Al, Si, K.
3. Sulphate precipitation: several elements (Ba, Pb, Sr) form insoluble sulphate precipitates, which will be filtered out from the leachate.

It is noteworthy that certain elements (e.g. Ba) form insoluble sulphates and therefore remain in the pulp tailings. Their removal from the uranium solution stream at the leaching stage can also be possibly used as a process indicator, as their concentration in the intermediate and final product will be very low if sulphuric acid is used during the process. The removal of insoluble sulphates is also advantageous for the age determination based on the ^228^Th/^232^Th chronometer, where the complete radium separation from thorium is the major condition for use [12]. The ^230^Th separation from its parent nuclide, the ^234^U, is ineffective, so the ^230^Th/^234^U dating tool cannot be used at this stage.

**Fig. 2:**
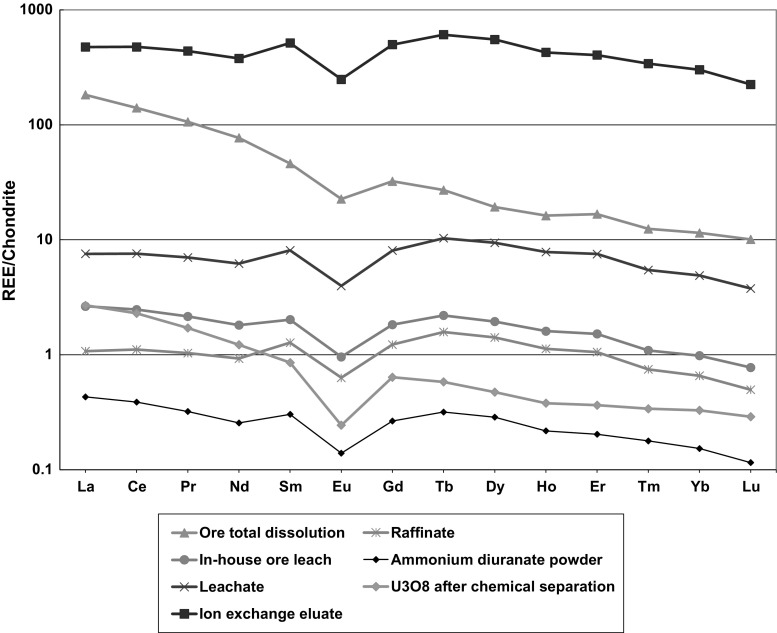
Rare-earth patterns of the investigated samples. The ion exchange eluate, ADU powder and U_3_O_8_ results were normalized to the uranium content before chondrite normalization, for the other samples the rare-earth concentration relative to the total sample weight was used

It is noted also that the REE pattern compared to the total ore REE content changes to some extent during the leaching step (Fig. 2). Especially the slope of the light REE side of the pattern is different: in the ore the light rare-earth elements are more abundant than in the leachate. This is attributed to the presence of not easily leachable minerals with higher light REE abundance. The overall pattern, however, and the Eu-anomaly, which is the other important characteristic of the REE pattern for the sampled materials, is largely unaltered. The REE pattern resembles quartz-pebble conglomerate UOC samples analysed previously [13]. This further underlies the applicability of REE pattern for the source (e.g. deposit-type) assessment.

The comparison of the in-house leach sample with the cleared leachate can help identify a few elements, which are possibly affected also by the process: the in-house leach sample contained significantly less Mn than the sampled leachate, so it was assumed that at the plant MnO_2_ was added as an oxidant, which increased the Mn-level in the leachate. This assumption was later confirmed by the plant operator. The increased levels of Na and Cd are possibly related to process-related contamination, while the higher levels of certain transition elements (Nb, Zr, Ti) are either due to the precipitation and instability of these elements in the cleared solutions after leaching or (less probably) due to the better extraction efficiency of the likely higher concentration of the sulphuric acid used for leaching at JRC-Karlsruhe (based on the sulphur results). The effect of recycled raffinate from the solvent extraction cannot be interpreted, because not enough data is available about the relative amounts of stream flows.

Analysis results for U isotopics are summarized in Supplement 2. All samples show natural composition: (*n*(^235^U)/*n*(^238^U) is (7.256 ± 0.016) × 10^−3^); *n*(^236^U)/*n*(^238^U) is below detection limit of about 4 × 10^−7^. The *n*(^234^U)/*n*(^238^U) slightly decreases from the feed ore towards the final product, but not significantly. The measured *n*(^234^U)/*n*(^238^U) interval overlaps with the reported values for quartz-pebble conglomerate [14]. Due to the high variability in ore, *n*(^234^U)/*n*(^238^U) can be used as a comparative signature due to its high variation.

**Fig. 3:**
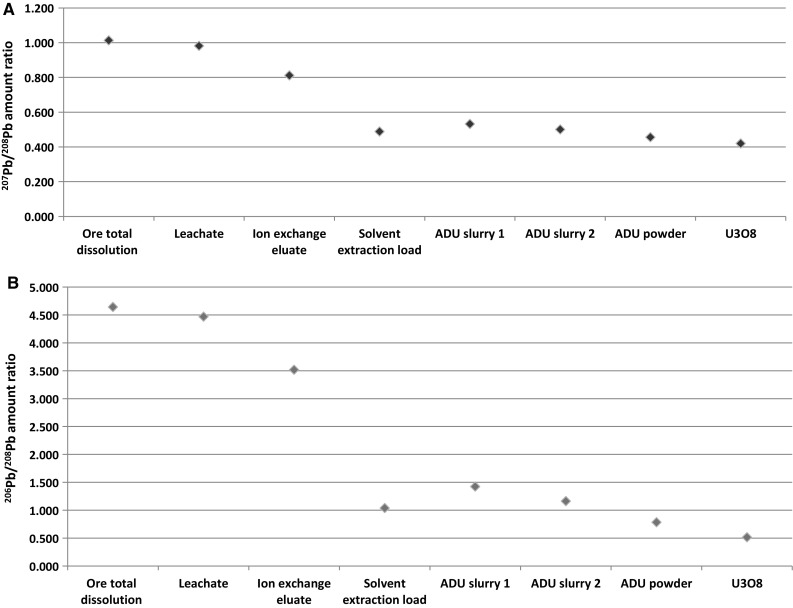
Pb isotopic ratios of the investigated samples. The measurement uncertainties are negligible, therefore *error bars* are not visible

**Fig. 4:**
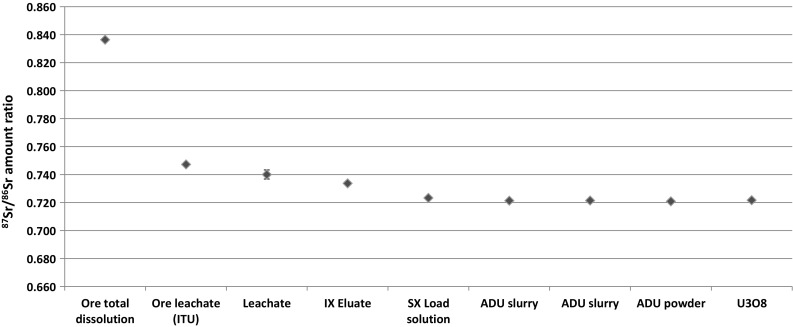
Sr isotopic ratios of the investigated samples

Isotopics of Pb and Sr are also changed throughout the process, especially during the leaching and extraction stages (Figs. 3, 4). Small variation can be explained by inhomogeneity of the samples (especially of the ore), but most of the difference comes from the H_2_SO_4_ leachable minerals, which is more pronounced for Sr (Fig. 4). Therefore, Sr and Pb isotopics in the leachate are not characteristic to the ore, and cannot be used to trace the processed material back to the ore. The (^207^Pb/^206^Pb)_radiogenic_ ratio is 0.078 ± 0.001, which can be used to assess the age of the ore body by the Pb–Pb method [10]. The derived age of 1600 ± 300 Ma years is in good agreement with the literature value of 1800 Ma years for this type of ore. This means that the Pb–Pb age can be used to identify (or exclude) certain possible sources.

**Fig. 5:**
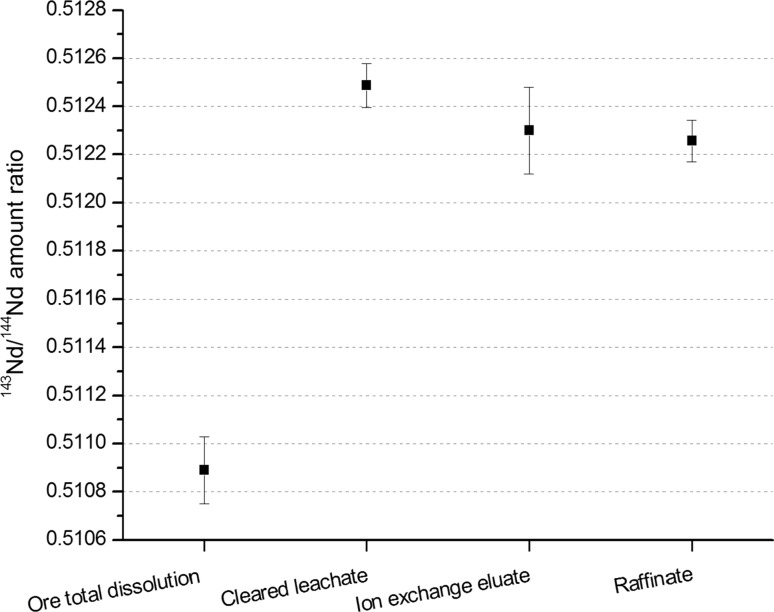
Nd isotopic ratios of the investigated samples

It was noted also that the Nd isotopic ratio changes during the dissolution if H_2_SO_4_ is used, and does not in the case of total dissolution (0.512488(91) and 0.51089(14), respectively) (Fig. 5 and Supplement 2). This indicates that with the H_2_SO_4_ only certain Nd-containing minerals will be leached, and thus not the average Nd isotopic ratio of the ore is measured.

**Fig. 6:**
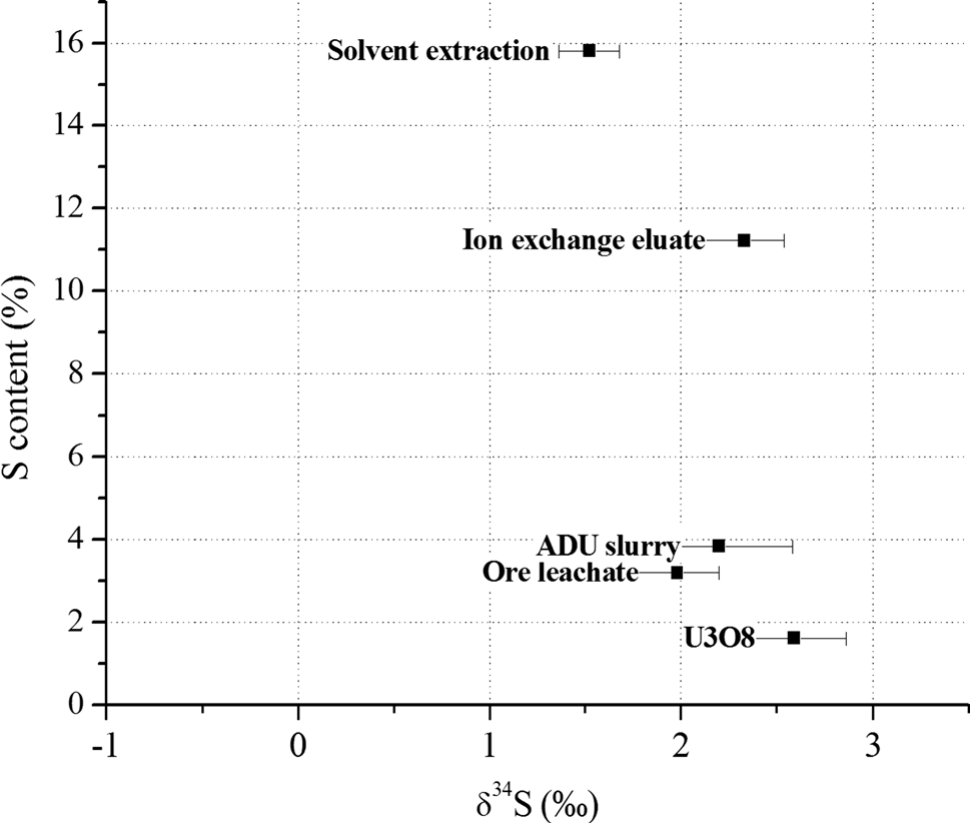
S isotopic ratios of the investigated samples together with the S content

The S isotopics and S content are shown in Fig. 6. It has been earlier demonstrated that most of the S (about 80%) derives from the H_2_SO_4_ reagent used for dissolution, thus it is process-indicative signature (i.e. it can potentially be used to verify for a reagent source if such information is available) [1, 15].

To sum up, we can conclude that the leaching/dissolution step has a radical effect on the impurity content and pattern. Even the chemically similar REEs fractionate slightly. The removal of several alkali earth elements is important (especially Ba), as it can be indicative of sulphuric acid leaching in comparison to carbonate-based processes. It also proves that high Ra/Th separation factor is expected for such materials, which is the prerequisite for the use of the ^228^Th/^232^Th chronometer. The Sr and Pb isotopics variation occur at this stage, this is attributable to the fractionation occurring during leaching of the ore minerals having different chemical and isotopic composition. The effect of process contamination, i.e. contribution from process chemicals and equipment, is most possibly negligible for Pb, Sr, Nd and REEs due to the high concentration levels in the ore. In case of S isotopics the sulphuric acid (98%) has obviously very high sulphur contribution even though the ore itself also contains high amount of sulphur (approximately 8400 μg g^−1^). Thus after this stage the sulphur isotopic composition will be between that of the ore and of the used H_2_SO_4_ reagent (closer to the acid). Though relative amounts are not known, and also the leaching efficiency of sulphur from the ore cannot be estimated without the knowledge of relative amounts used, we can expect that the sulphur isotopic composition after this stage will be closer to that of the process chemicals (sulphuric acid at this stage, other sulphates later) due to their presumably higher quantity.

### Step 2: Ion exchange separation

After the ore leaching stage, the leachate is cleared in thickeners, and the cleared leachate is purified using ion exchange (IX) separation. 12% sulphuric acid is used as eluent.

The measured concentration results are given in Supplement 1 and Table 3. A separation factor was calculated for each element defined as the ratio of the concentration in the cleared leachate and the concentration in the IX eluate (all values normalized to uranium). The higher this separation factor, the better the efficiency of uranium separation from the element concentrated in the ion exchange separation step. This is theoretically inversely proportional to the distribution coefficient of the analyte of interest to that of uranium (uranyl) in sulphuric acid medium.

**Table 3:** Impurity concentrations in the IX eluate and their ratios relative to that of the cleared leachate (separation factor)

	IX eluate concentrations in μg g^−1^ (±uncertainty)	Ratio cleared leachate to IX eluate		IX eluate concentrations in μg g^−1^ (±uncertainty)	Ratio cleared leachate to IX eluate
Ag	Less than 1.0		Na	38300 ± 2200	38.9
Al	45,700 ± 2600	*133*	Nb	4.9 ± 0.3	**3.8**
As	11,500 ± 670	16.0	Nd	171.3 ± 9.9	92.0
Au	15.2 ± 0.9		Ni	1652 ± 96	*113*
B	57.3 ± 3.3		Os	Less than 0.7	
Ba	18.3 ± 1.1	***0.7***	P	6670 ± 390	21.4
Be	5.3 ± 0.3	*168*	Pb	443 ±	73.4
Bi	22.5 ± 1.3	**3.5**	Pd	18.6 ± 1.1	99.6
Ca	32,400 ± 1900	*109*	Pr	39.1 ± 2.3	89.6
Cd	8.6 ± 0.5	82.7	Pt	0.063 ± 0.004	
Ce	287 ± 17	89.3	Rb	22.8 ± 1.3	*118*
Co	869 ± 50	74.4	Re	0.22 ± 0.01	41.1
Cr	1034 ± 60	63.5	Ru	0.9 ± 0.1	
Cs	9.2 ± 0.5	96.4	S	11700000 ± 67900	**3.0**
Cu	861 ± 50	51.7	Sb	5.6 ± 0.3	85.3
Dy	133.9 ± 7.8	95.9	Sc	120.9 ± 7.0	**18.6**
Er	64.3 ± 3.7	*104*	Se	18.1 ± 1.0	*197*
Eu	13.9 ± 0.8	89.6	Si	64700 ± 3700	*113*
Fe	3,75,000 ± 22000	28.7	Sm	75.7 ± 4.4	88.3
Ga	10.5 ± 0.6	*122*	Sn	2.5 ± 0.1	
Gd	98.1 ± 5.7	90.7	Sr	154.9 ± 9.0	97.0
Ge	35.4 ± 2.1	36.6	Ta	2.2 ± 0.1	
Hf	6.3 ± 0.4	**13.3**	Tb	22.1 ± 1.3	95.3
Hg	Less than 0.5		Te	3.7 ± 0.2	21.7
Ho	23.7 ± 1.4	*103*	Th	13800 ± 800	**8.9**
In	0.77 ± 0.04	45.5	Ti	208 ± 12	**8.1**
Ir	0.063 ± 0.004		Tl	0.9 ± 0.1	*118*
K	8600 ± 500	94.2	Tm	8.2 ± 0.5	90.0
La	112 ± 6	89.1	V	105.6 ± 6.1	75.3
Li	50.7 ± 2.9	*146*	W	10.1 ± 0.6	
Lu	5.5 ± 0.3	94.4	Y	441 ± 26	*104*
Mg	27,000 ± 1600	93.6	Yb	49.0 ± 2.8	91.0
Mn	32,200 ± 1900	*166*	Zn	1960 ± 110	*142*
Mo	91.2 ± 5.3	**1.7**	Zr	434 ± 25	**1.1**

All concentration results are given as microgram of analyte per gram of uranium in the sample. Elements with very high separation factors (above 100) are highlighted. U concentration in the IX eluate was (3210 ± 48) μg g^−1^

Low separation factors were found for the sulphate anion complex-forming elements (e.g. Mo, Nb, Th, Zr), indicating that, similarly to uranium, anion complexes of these elements have high retention on the anion exchange resin, thus less separation is expected. The higher abundance of such elements in the intermediate product can be a potential signature to discriminate anion exchange purification processes, as these separation factors should correlate with the theoretical distribution coefficient on the anion exchange resin in sulphate medium. In case of Ba the ineffective separation is attributed also to its virtually complete removal in the leaching stage due to the BaSO_4_ precipitation.

The REE pattern is mainly unaltered in the ion exchange separation (Fig. 2), which is also in agreement with our previous findings [13]. U isotopics are unaltered in this step. Sr, Pb and Nd are effectively removed by ion exchange separation, but no isotopic fractionation is expected during this step, as Sr, Pb and Nd are not added intentionally. The statistical significant alteration of Sr and Pb isotope ratios are possible due to the contribution of the chemicals to the feed, which is at low-level, but relatively high (compared to the impurity level) amount. Therefore, the isotopics of Sr and Pb are changed, and they cannot be used to trace the sampled material to the original ore. In case of *n*(^133^Nd)/*n*(^134^Nd) there is no significant change in this step (0.512488(91) and 0.51230(18), respectively), which means that there is no contribution of Nd from the reagents (Fig. 5).

The separation factor of sulphur (concentration change) includes also the contribution from the eluent (12% sulphuric acid), so probably the ore-derived sulphur content and isotopic composition is completely diminished at this stage [16].

The Th/U separation factor is relatively low; therefore the ^230^Th/^234^U chronometer cannot be used to estimate the date of this ion exchange separation step [17]. However, for most elements, high separation factors (above 50) were observed, identifying this stage as an effective purification and pre-concentration step.

### Step 3: Solvent extraction

Following the ion exchange separation, uranium is further purified and pre-concentrated at the plant using solvent extraction (Alamine-336, three stages). Alamine-336 (registered trademark of BASF SE) is a water insoluble, tri-octyl/dodecyl amine which is capable of forming oil soluble salts of anionic species at low pH. For uranium stripping, ammonium sulphate is used. The feed is the eluate from IX separation. Both the purified solution (the resulted loaded solution, called OK liquor) and the raffinate were sampled. The results are summarized in Supplement 1 and Table 4.

**Table 4:** Relative impurity concentrations and separation factors during the solvent extraction step

	Ratio IX eluate to loaded solution (separation factor)	Ratio IX eluate versus raffinate		Ratio IX eluate to loaded solution (separation factor)	Ratio IX eluate versus raffinate
Ag	NA		Na	6.0	1.85E+01
Al	13,500	2.87E−02	Nb	**1.5**	
As	221	2.89E+00	Nd	7750	5.28E−02
Au	**6.5**	3.72E+01	Ni	Higher than 680	
B	4.5		Os	NA	
Ba	17.3	1.72E+01	P	944	4.42E−01
Be	Higher than 8.5		Pb	Higher than 4020	
Bi	**7.9**	5.45E+01	Pd	120	3.33E+00
Ca	11.4	2.57E+01	Pr	Higher than 10^5^	
Cd	24.4	2.39E+01	Pt	**1.9**	
Ce	13,000	3.30E−02	Rb	19	1.67E+01
Co	**2.8**	1.61E+02	Re	**4.0**	4.42E+01
Cr	1200	2.78E−01	Ru	Higher than 1070	
Cs	34.1	1.10E+01	S	**2.5**	1.95E+02
Cu	Higher than 1070		Sb	51	9.20E+00
Dy	12,100	3.22E−02	Sc	450	1.01E+00
Er	Higher than 10^5^		Se	**0.7**	4.79E+01
Eu	Higher than 10^5^		Si	Higher than 180	
Fe	9430	5.23E−02	Sm	6900	5.90E−02
Ga	633	5.19E−01	Sn	16	
Gd	8880	4.59E−02	Sr	52	6.80E+00
Ge	92		Ta	399	
Hf	**1.8**	5.31E+02	Tb	Higher than 10^5^	
Hg	NA		Te	1.4	
Ho	Higher than 10^5^		Th	5170	8.38E−02
In	Higher than 10^5^		Ti	143	2.79E+00
Ir	**1.3**		Tl	85	2.66E+00
K	16	2.46E+01	Tm	Higher than 1e5	
La	281	1.58E+00	V	Higher than 442	
Li	42	9.11E+00	W	**6.7**	8.11E+01
Lu	Higher than 10^5^		Y	11400	3.50E−02
Mg	16	2.77E+01	Yb	Higher than 10^5^	
Mn	208	1.49E+00	Zn	258	2.19E+00
Mo	**2.7**	3.11E+02	Zr	**1.5**	7.72E+02

U concentration in the raffinate was (247.8 ± 3.0) μg g^−1^. Elements close to 1 are highlighted

*NA* not applicable

A separation factor was also calculated for each element in the same way as for the ion exchange separation, defined as the ratio of the normalized element concentration in the IX eluate (feed material for this step) and the normalized element concentration in the loaded solution (product). All values were normalized to uranium for calculating the separation factors. The higher this separation factor, the better the efficiency of uranium separation from the element concerned in the solvent extraction step. This is theoretically inversely proportional to the similarity of chemical behaviour of the analyte of interest to uranium (uranyl) during the solvent extraction stage.

Relatively low separation factors were found for several elements (indicated in Table 4), such as Au, Bi, Co, Hf, Ir, Mo, Nb, Pt, Re, S, Se, Te, W or Zr. The possible reason for this finding is that the extraction nature of Alamine-336 is based on ion exchange reaction of the protonated amine with anions (in case of uranium separation with the anionic uranyl sulphate complex). The schematic reaction is shown below:

1 $$2{\text{R}}_{3} {\text{N}} + {\text{H}}_{2} {\text{SO}}_{4} = \left( {{\text{R}}_{3} {\text{NH}}} \right)_{2} {\text{SO}}_{4}$$

2 $$2 { }\left( {{\text{R}}_{ 3} {\text{NH}}} \right)_{ 2} {\text{SO}}_{ 4} + {\text{UO}}_{ 2} \left( {\text{SO}} \right)_{ 3}^{ 4- } = \left( {{\text{R}}_{ 3} {\text{NH}}} \right)_{ 4} {\text{UO}}_{ 2} \left( {{\text{SO}}_{ 4} } \right)_{ 3} + 2 {\text{ SO}}_{ 4}^{ 2- }$$

Elements which are present as anions (e.g. Re, Mo or Se) or form anionic sulphate complexes (e.g. Zr, Hf), will take part in the reactions given above, and in consequence have lower separation factors, i.e. they are less separated from uranium. Note that relatively low separation factors were observed in solvent extraction for mainly the same elements as for the ion exchange step, which can be explained by the fact that the separation mechanism in both cases are related to ion exchange. Notable differences are Sc, Ti or Th, where the solvent extraction is more effective than the ion exchange (less stable sulphate complexes are formed during the solvent extraction), thus they may serve as discriminator between the anion exchange as in Step 2 and the amine-based solvent extraction processes.

The S concentration change was found to be relatively low, meaning that sulphur from the previous stages is assumed to have exchanged continually with the sulphur from the process chemicals, since sulphate is added in high amount to the purified material also at this stage (ammonium sulphate solution is used as a stripping agent). Pb and Sr are very effectively separated also at this stage and in the eluate they reach very low concentration levels, comparable to the typical concentration values in the industrial chemicals used. This implies that from this step onwards the Pb or Sr contribution of the process chemicals will alter the Pb or Sr isotopic composition. Nd could not be determined due to its low concentration.

Th is well separated from uranium at the SX stage, so if production date of the material is determined by the Th/U radiochronometry, the measured production date will refer to this step of the UOC production. The total Th/U separation factor of combining both the ion exchange and the solvent extraction steps is 7 × 10^7^. According to our previous study this residual thorium level can cause only approximately 0.5% positive bias for a 1-year-old material using the ^230^Th/^234^U chronometer for dating studies [12], which implies that one can obtain accurate age dating results.

The complete REE pattern of the loaded solution could not be established with the standard method due to the low concentration of heavy REEs. However, as the REE pattern of the raffinate is identical to that of the IX eluate, it is expected that the REE pattern is not altered by this type of amine-based solvent extraction (Fig. 2). This was verified later by the REE analysis (performed after a group REE separation from uranium) of the purified products (ADU slurries and U_3_O_8_). The robustness of the REE pattern during the solvent extractions using amine-based extractants was also anticipated previously [13]. Similarly to the REE pattern, in the future it would be useful to investigate the patterns of some of the platinum-group elements (PGE: Os, Ir, Ru, Rh, Pt, Pd). As these elements are very abundant in the investigated ore deposit and they are also retained on measurable levels in the purified uranium products, they could be potential signatures. These elements behave chemically relatively similar (e.g. the separation factors of Ir and Pt are 1.3 and 1.9, respectively), so they are less prone to the fractionation due to the metallurgical process and thus their ratio values (or patterns) can be characteristic of the given ore deposit. In order to use the PGE, better detection limits are required (most possibly achievable through chemical pre-concentration), as well as modification of the ICP-MS procedure (avoiding the use of Rh as internal standard).

**Fig. 7:**
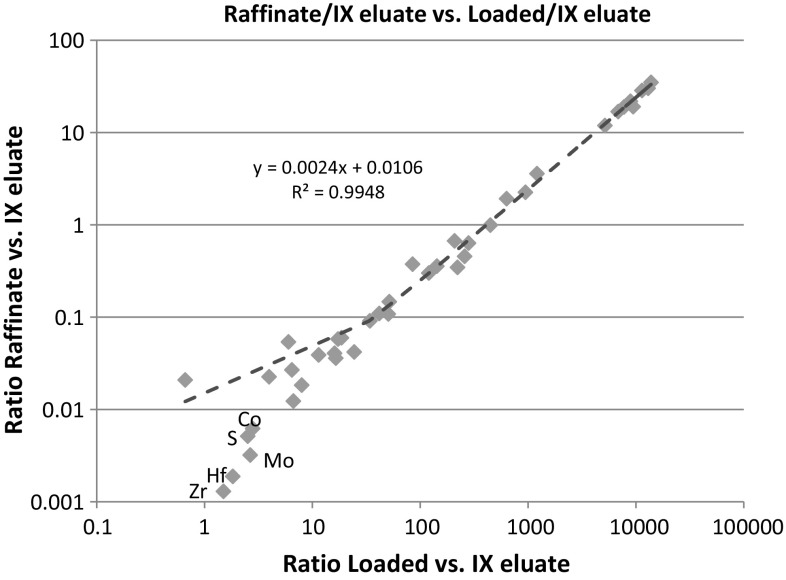
Concentration ratio of raffinate/IX eluate versus the concentration ratio of Loaded/IX eluate (concentration values are normalized to uranium). The *dotted curve* indicates the theoretical linear correlation

The separation factors (the ratio of the concentration relative to uranium in the loaded solution and the concentration in the IX eluate) are inversely proportional to the ratio of the concentration of the element of interest in the raffinate relative to the IX eluate, i.e. the higher the separation factor is, the higher portion of the element remains in the raffinate, while uranium is extracted to the organic phase. If we plot then the concentration ratio of raffinate/IX eluate versus the concentration ratio of Loaded solution/IX eluate, we should get a linear correlation (Fig. 7). Any distortion from this relationship may be the result of the addition of other chemicals or due to process contamination. Significant differences (above 25%) were found for Bi, Co, Cd, Hf, Mg, Mo, Na, S, W, Zn and Zr (for the underlined ones the deviation is higher than 100%); in each case the measured concentration in the raffinate was *lower* than the theoretical value. For most of these elements (Hf, Mo, W, Zr) this is possibly due to the precipitation and instability in the raffinate solution, thus these elements are likely present as solid residue in the raffinate. The reasons for other common elements being different from the theoretical estimate are either because the trace-level concentration in the loaded solution is higher due to process contamination, or because of the contribution from the barren mother liquor, which is recycled back to the solvent extraction from the forthcoming ADU precipitation step.

### Step 4: Precipitation

Uranium is precipitated at the plant from the loaded solution using ammonia as ammonium diuranate (ADU). Both the ADU slurry and the barren mother liquor were sampled. The barren solution is recycled back to the solvent extraction step.

The impurity concentration results are summarized in Supplement 1. Similarly to the methodology applied before, a separation factor can be calculated for each analyte defined as the concentration ratio of the loaded solution (starting material) relative to that in the ADU slurry (precipitate). The separation factors are summarized in Table 5. If this separation factor value is higher than 1.25 (arbitrarily chosen), the impurity concerned is separated (removed) significantly from uranium during the ADU precipitation. A separation factor value lower than 0.75 means that the concentration of the analyte concerned increased significantly in the precipitate, i.e. the corresponding impurity concentrates in the uranium material. A 25% threshold was chosen to rule out possible effects of inhomogeneity. Thus, a separation factor between 0.75 and 1.25 indicates a quantitative precipitation (no separation effect). Note that the separation factor approach calculates the *overall* change of the impurity concentration for this step. The concentration change can also be the result of several simultaneous phenomena, e.g. a concentration decrease by chemical separation and a parallel concentration increase due to process contamination from the chemicals used.

**Table 5:** Variation of impurity concentrations during the precipitation step

	Ratio precipitate versus loaded solution (separation factor)	Barren mother concentrations in (μg g^−1^) (±uncertainty)		Ratio precipitate versus loaded solution (separation factor)	Barren mother concentrations in μg g^−1^ (±uncertainty)
Ag		Less than 3.9E−02	Na	**4.43**	**1.22E**+**01** ± **7.1E**−**01**
Al	*0.039*	Less than 9.5E−01	Nb	*0.82*	Less than 9.8E−04
As	**1.51**	**3.38E**−**02** ± **2.0E**−**03**	Nd	*0.23*	Less than 1.4E−03
Au	**Higher than 186**	**1.06E**−**02** ± **6.2E**−**04**	Ni		Less than 8.5E−02
B	**1.50**	**8.08E**−**01** ± **4.7E**−**02**	Os		Less than 5.3E−02
Ba	*0.34*	Less than 4.6E−03	P	1.05	Less than 2.3E+00
Be		Less than 5.1E−02	Pb		Less than 2.0E−02
Bi	**1.89**	**5.20E**−**03** ± **3.0E**−**04**	Pd	**Higher than 3**	**1.16E**−**03** ± **6.7E**−**05**
Ca	**1.41**	**9.52E**+**00** ± **5.5E**−**01**	Pr		Less than 9.0E−05
Cd	0.95	Less than 1.2E−03	Pt	1.16	Less than 8.1E−04
Ce	*0.11*	Less than 3.3E−04	Rb	1.10	Less than 2.4E−02
Co	**166**	**1.04E**+**00** ± **6.0E**−**02**	Re	**7.85**	**2.89E**−**04** ± **1.7E**−**05**
Cr	*0.39*	Less than 6.7E−03	Ru		Less than 4.4E−04
Cs		Less than 5.9E−03	S	**114**	**1.46E**+**04** ± **8.5E**+**02**
Cu		Less than 4.0E−02	Sb	0.81	Less than 5.0E−04
Dy	*0.70*	Less than 1.1E−04	Sc	0.86	Less than 1.4E−03
Er		Less than 9.6E−05	Se	**Higher than 12**	**2.64E**−**01** ± **1.5E**−**02**
Eu		Less than1.1E−04	Si		Less than 6.5E+01
Fe	*0.28*	Less than 3.9E−01	Sm		Less than 7.0E−04
Ga	*0.62*	Less than 2.5E−03	Sn	**Higher than 78**	Less than 1.7E−02
Gd	*0.36*	Less than 2.5E−04	Sr	*0.32*	**9.57E**−**04** ± **5.5E**−**05**
Ge	**Higher than 1.4**	Less than 1.0E−01	Ta		Less than 3.7E−03
Hf	0.96	Less than 2.1E−04	Tb		Less than 4.1E−05
Hg		Less than 3.2E−02	Te	1.15	Less than 5.1E−03
Ho		Less than 2.9E−05	Th	0.84	Less than 6.1E−04
In		Less than 1.0E−03	Ti	*0.54*	Less than 1.3E−02
Ir	**1.29**	Less than 8.5E−03	Tl		Less than 5.0E−04
K	**1.82**	**1.87E**+**00** ± **1.1E**−**01**	Tm		Less than 3.7E−05
La	*0.81*	Less than 5.0E−04	V		Less than 8.3E−03
Li	**Higher than 1.8**	Less than6.3E−02	W	0.92	Less than 3.3E−03
Lu		Less than 2.7E−05	Y	*0.32*	Less than 7.2E−04
Mg	**3.57**	**4.88E**+**00** ± **2.8E**−**01**	Yb		Less than 5.8E−05
Mn	**14.2**	**7.72E**−**01** ± **4.5E**−**02**	Zn	**1.41**	Less than 4.8E−01
Mo	1.13	**1.14E**−**03** ± **6.6E**−**05**	Zr	0.83	**5.49E**−**03** ± **3.2E**−**04**

Concentration ratios in the precipitate (ADU slurry-1) relative to that of in the loaded solution (separation factor)

Barren mother concentration results in microgram analyte per gram U in the sample are also indicated. Separation factors higher than 1.25, less than 0.75 and between 0.75 and 1.25 are highlighted

Several elements are effectively and significantly removed during the ADU precipitation step; these include As, **Au**, B, Bi, Ca, **Co**, Ge, Ir, K, Li, Mg, **Mn**, **Na**, Pd, **Re**, **S**, **Se**, **Sn**, Zn. The elements with separation factors higher than 4 are indicated in bold; they remain predominantly in the aqueous phase and separated from the solid ADU precipitate. Several of these elements are soluble in ammonia solution and do not form insoluble precipitates (e.g. As, Na or S as sulphate, Se), others form soluble ammonia complexes (Zn or Co, which is possibly present as Co^3+^, forms stable hexamine complex), or soluble hydroxide complexes (Sn or Au possibly as Au(OH)_4_^−^) the presence of excess ammonia. A similarly high separation factor is expected for a few elements forming strong amine complex (such as Hg, Cu or Ag), which were below detection limit in the loaded solution or in the precipitate. The relatively high separation of Mn is not fully understood, as it should form an insoluble precipitate (Mn(OH)_2_ or MnO_2_.H_2_O) and co-precipitate with uranium.

Concentrations of several elements increased significantly during the ADU precipitation stage (i.e. separation factors were significantly lower than 1); these include Al, Ba, Ce, Cr, Dy, Fe, Ga, Gd, In, Nd, Sr and Ti. For the commonly occurring elements this increase is attributed mainly to the process contamination (i.e. addition from the chemicals used or from erosion/corrosion of the production lines and vessels). The significant increase of REE concentrations is not fully understood: either it is a measurement problem due to the very low concentrations, or it is related to the recycling of the barren solution to the solvent extraction step, and thus a higher contribution from a previous production batch. The recycled barren mother solution can also contribute to the concentration increase of the other common elements (e.g. Al, Ba, Cr, Fe, Sr or Ti).

Production date of one of the ADU slurry-2 was determined using the ^230^Th/^234^U method. The obtained result (9 March 2013 with an uncertainty of 56 days) is in good agreement with the known production date (12 February 2013).

### Step 5: Drying and calcination

**Fig. 8:**
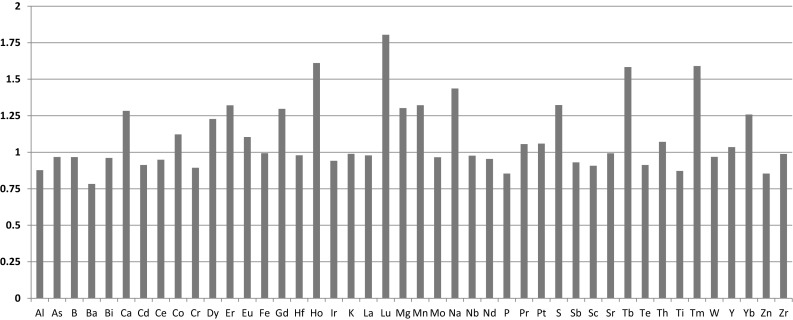
Impurity concentration ratio of ADU slurries

At the final stage, the ADU slurry is dried and calcined to U_3_O_8_. All samples except the dry ADU powder were taken directly from the process line. The ADU powder was sampled at the plant analytical laboratory and represents a material processed 2–3 weeks earlier. The ADU slurry was sampled twice. The impurity concentration ratios of two slurries are shown in Fig. 8. For most impurity elements, the concentration ratios in the two ADU slurry samples are close to 1 with a few exceptions indicating the almost identical composition of the samples. Exceptions, where this deviation is higher than 25% are Ca, Mg, Mn, Na, S and surprisingly the heavy REEs (Gd–Lu). For the transition metals and sulphur this observation is possibly related to the variations between different batches (more likely) or sample inhomogeneity (less likely). For the heavy REEs it could be a measurement issue due to the very low concentrations (the ADU slurries were analysed with the standard procedure without chemical pre-concentration) and the underestimation of overall uncertainties. The REE pattern of ADU powder and U_3_O_8_ is the same indicating the same source (Fig. 2).

**Fig. 9:**
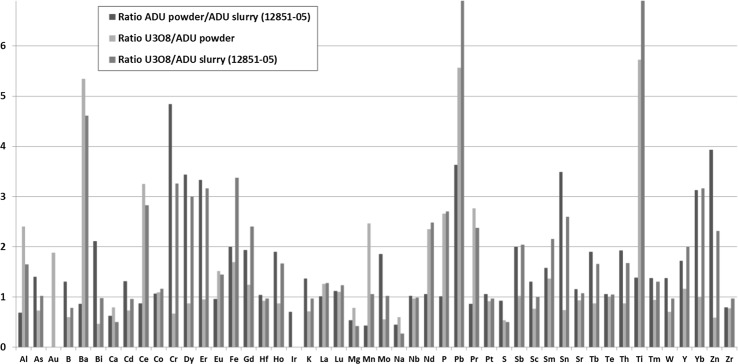
The impurity concentration ratios of the ADU powder and ADU slurry-2, U_3_O_8_ product and ADU powder, U_3_O_8_ product and ADU slurry-2

At the plant, ADU slurry is dried in a low temperature process by rotating vacuum filters, while calcination takes place under more vigorous conditions at 490 °C for 6 h, thus a bigger effect on the impurity pattern can be expected at the latter step. The impurity concentration ratios of the ADU powder relative to ADU slurry-2 and the impurity concentration ratios of the U_3_O_8_ product relative to ADU powder are shown in Fig. 9. Note that the variation reflects not only the change due to the process but (for most metallic impurities) also the difference between the batches produced. Based on the data available it is reasonable to assume that sulphur is mostly eliminated in the calcination stage, similarly to other volatile components. Depending on the calcination temperature and conditions this decrease might be explained by the volatility of certain elements (such as B, Cd or Hg) at elevated temperatures, though from the present dataset it cannot be concluded with high confidence.

The behaviour of Pb and its continuous concentration increase appears to be a consequence of the process-related contamination, i.e. Pb impurity is inadvertently introduced via chemicals used. Note that Pb was very effectively separated at the ion exchange and solvent extraction stages to a very low concentration level, below 0.1 μg/gU. Input of Pb from reaction vessels in the forthcoming drying and calcination step of this commonly occurring element may be a straightforward explanation for the increase. This assumption can be verified in the future by the measurement of the Pb isotopic composition, as the ^204^Pb, ^206^Pb, ^207^Pb and ^208^Pb abundances are 1.4, 24.1, 22.1 and 52.4%, respectively, in most process-related vessels and materials, while uranium ores have highly radiogenic lead isotopic composition. By the increase of the process contamination contribution, the radiogenic Pb isotopic composition from the uranium ore approaches to that of the commonly occurring lead isotopic composition. Though the ADU powder sample was not taken directly from the material flow, its REE pattern is identical to those of the intermediate products (Fig. 2). This indicates the robustness and applicability of the REE pattern to verify the source of the feed material.

**Fig. 10:**
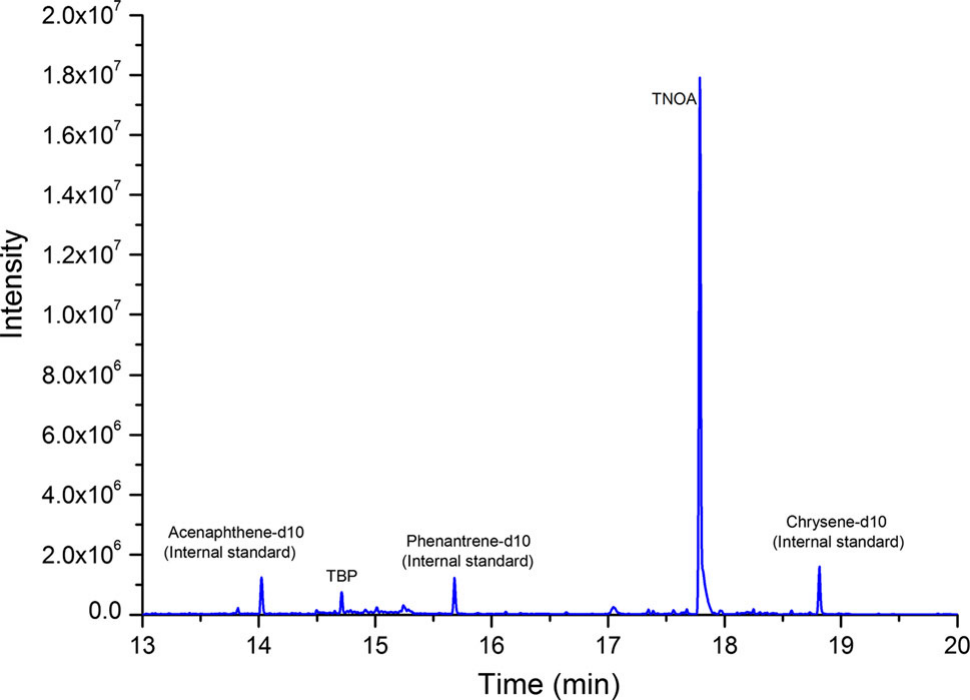
GC-MS chromatogram of the ADU powder

Among the target organic impurities, TNOA was positively identified in the measured chromatogram of the ADU powder, while the measured TBP and TOPO concentrations were low (Fig. 10); this confirms the use of the amine-process (solvent extraction) during the ADU production. The TNOA concentration was high, about 700 ng g^−1^, the estimated relative uncertainty is about 15%. Note that the emphasis was on the detection of the target analytes rather than on the precision of the GC–MS measurement, as the concentration of organic residues is expected to be highly variable due to the several process stages following the solvent extraction. The organics were not measureable in the calcined U_3_O_8_ product, as they oxidize and decompose during the calcination.

The production date of U_3_O_8_ sample was determined based on the ^230^Th/^234^U method. The obtained result (1 February 2013 with an uncertainty of 74 days) is in good agreement with the known production date (14 February 2013).

## Conclusions

Uranium ore concentrate (UOC) production from ore until U_3_O_8_ was followed by the systematic sampling and measurement of various key parameters. It was found that for this specific ore and particular process the REEs, certain impurities, Pb–Pb deposit age, production date after SX step and organic measurements were relevant, whereas isotopics of Sr, Pb and concentration changes of some impurities in the processed material can be misleading. Similarly to the REE pattern, in the future it would be useful to investigate the patterns of some of the platinum-group elements (PGE): Os, Ir, Ru, Rh, Pt, Pd. Although the methodology refers to a specific UOC production process, one can generalize it and identify several signatures useful both in nuclear safeguards and forensics.

## Electronic supplementary material

Below is the link to the electronic supplementary material.

Supplementary material 1 (DOCX 174 kb)
