# Supplementary material for: Identification of uranium signatures relevant for nuclear safeguards and forensics
Source: J Radioanal Nucl Chem. 2017 Apr 20;312(3):639–54. doi: 10.1007/s10967-017-5247-5 (PMC5446562; doi:10.1007/s10967-017-5247-5)
Supplement: Supplementary file 1 — Supplementary material 1 (DOCX 174 kb) [file 10967_2017_5247_MOESM1_ESM.docx]

**Supplement 1.**

Uncertainties are the total combined uncertainties with a coverage factor of 1. If no result is given, the value is below the quantification limit. The value stated as *U* is then the actual quantification limit.

**Supplement 2.**

|  | **^87^Sr/^86^Sr amount ratio** | **Uncertainty (*k*=2)** |
| --- | --- | --- |
| Ore total dissolution | 0.83646 | 0.00030 |
| Ore leachate (ITU) | 0.74733 | 0.00029 |
| Leachate | 0.74010 | 0.00023 |
| IX Eluate | 0.73372 | 0.00021 |
| SX Load solution | 0.7234 | 0.0033 |
| ADU slurry-1 | 0.72136 | 0.00051 |
| ADU slurry-2 | 0.72148 | 0.00056 |
| ADU powder | 0.72089 | 0.00026 |
| U_3_O_8_ | 0.72170 | 0.00018 |

|  | **^207^Pb/^208^Pb amount ratio** | **U (*k*=2)** | **^206^Pb/^208^Pb amount ratio** | **U (*k*=2)** | **^204^Pb/^208^Pb amount ratio** | **U (*k*=2)** |
| --- | --- | --- | --- | --- | --- | --- |
| Ore total dissolution | 1.01382 | 0.00070 | 4.6442 | 0.0026 | 0.02391 | 0.00029 |
| Leachate | 0.98149 | 0.00053 | 4.4700 | 0.0036 | 0.0239 | 0.0011 |
| Ion exchange eluate | 0.81167 | 0.00044 | 3.5182 | 0.0017 | 0.02213 | 0.00022 |
| Solvent extraction load | 0.4891 | 0.0051 | 1.0405 | 0.0089 | 0.0259 | 0.0063 |
| ADU slurry 1 | 0.5326 | 0.0041 | 1.425 | 0.010 | 0.0262 | 0.0039 |
| ADU slurry 2 | 0.5011 | 0.0057 | 1.1629 | 0.0085 | 0.0260 | 0.0055 |
| ADU powder | 0.45653 | 0.00032 | 0.78569 | 0.00044 | 0.02621 | 0.00033 |
| U_3_O_8_ | 0.42018 | 0.00032 | 0.51731 | 0.00047 | 0.02630 | 0.00035 |

|  | **^235^U/^238^U amount ratio** | **U (*k*=2)** | **^234^U/^238^U amount ratio** | **U (*k*=2)** | **^236^U/^238^U amount ratio** | **U (*k*=2)** |
| --- | --- | --- | --- | --- | --- | --- |
| **Ore total dissolution** | 0.007243 | 0.000029 | 0.00005396 | 0.00000083 | < LOD | < LOD |
| **Leachate** | 0.007258 | 0.000040 | 0.00005342 | 0.00000074 | < LOD | < LOD |
| **Ion exchange eluate** | 0.007289 | 0.000020 | 0.00005372 | 0.00000058 | < LOD | < LOD |
| **Solvent extraction load** | 0.007227 | 0.000061 | 0.00005313 | 0.00000094 | < LOD | < LOD |
| **ADU slurry 1** | 0.007274 | 0.000014 | 0.00005334 | 0.00000066 | < LOD | < LOD |
| **ADU slurry 2** | 0.007254 | 0.000016 | 0.00005316 | 0.00000067 | < LOD | < LOD |
| **ADU powder** | 0.007255 | 0.000025 | 0.00005288 | 0.00000081 | < LOD | < LOD |
| **U_3_O_8_** | 0.007245 | 0.000022 | 0.00005280 | 0.00000068 | < LOD | < LOD |

|  | **^143^Nd/^144^Nd amount ratio** | **U (*k*=2)** |
| --- | --- | --- |
| **Ore total dissolution** | 0.51089 | 0.00014 |
| **Leachate** | 0.512488 | 0.000091 |
| **Ion exchange eluate** | 0.51230 | 0.00018 |
| **Raffinate** | 0.512257 | 0.000086 |
